# A Compact MIMO Antenna Based on Modal Analysis for 5G Wireless Applications

**DOI:** 10.3390/mi15060729

**Published:** 2024-05-30

**Authors:** Parveez Shariff Bhadravathi Ghouse, Deepthi Mariam John, Pallavi R. Mane, Debdeep Saha, Supreetha Balavalikar Shivarama, Sameena Pathan, Bharathi Raghavendra Bhat, Shweta Vincent, Tanweer Ali

**Affiliations:** 1 Department of Electronics and Communication Engineering, Manipal Institute of Technology, Manipal Academy of Higher Education, Manipal 576104, India; parveez.bg@learner.manipal.edu (P.S.B.G.); deepthi.john1@learner.manipal.edu (D.M.J.); supreetha.bs@manipal.edu (S.B.S.); 2 Department of Electrical and Electronics Engineering, Manipal Institute of Technology, Manipal Academy of Higher Education, Manipal 576104, India; debdeep.saha@manipal.edu (D.S.); bharathi.rb@manipal.edu (B.R.B.); 3 Department of Information and Communication Technology, Manipal Institute of Technology, Manipal Academy of Higher Education, Manipal 576104, India; 4 Department of Mechatronics, Manipal Institute of Technology, Manipal Academy of Higher Education, Manipal 576104, India; shweta.vincent@manipal.edu

**Keywords:** 5G, CMT, dipole array antenna, millimeter wave (mmWave), metasurface, MIMO

## Abstract

This article presents a planar, non-angular, series-fed, dual-element dipole array MIMO antenna operating at 28 GHz with the metasurface-based isolation improvement technique. The initial design is a single-element antenna with a dual dipole array which is series-fed. These dipole elements are non-uniform in shape and distance. This dipole antenna results in end-fire radiation. The dipole antenna excites the $J_{1}$ mode for its operation. Further, with the view to improve channel capacity, the dipole array expands the antenna to a three-element MIMO antenna. In the MIMO antenna structure, the sum of the $J_{1}$, $J_{2}$, and $J_{3}$ modes is excited, causing resonance at 28 GHz. This article also proposes a metasurface structure with wide stopband characteristics at 28 GHz for isolation improvement. The metasurface is composed of rectangle-shaped structures. The defected ground and metasurface structure combination suppresses the surface wave coupling among the MIMO elements. The proposed antenna results in a bandwidth ranging from 26.7 to 29.6 GHz with isolation improvement greater than 21 dB and a gain of 6.3 dBi. The antenna is validated with the diversity parameters of envelope correlation coefficient, diversity gain, and channel capacity loss.

## 1. Introduction

The millimeter-wave spectrum is explored in the field of communication [1], including in vehicular [2] and biosensors [3] applications, due to its short distance, broad bandwidth, and low-latency features. An additional boost in communication performance is achieved with the multiple-input multiple-output (MIMO) technique [4]. It ensures better signal quality through spatial multiplexing in a fading multipath channel [5]. Numerous state-of-the-art MIMO antennas exist [6,7,8,9]; however, this article focuses on dipole antennas, as these are desirable for future handheld devices. The dipole antenna is a simple and compact mmWave structure, and the desired resonance is simply achieved by tuning the dipole length. The series of uniform or non-uniform array structures further improves the gain and bandwidth of the dipole antenna [10,11,12]. A balun can also be used for better impedance matching at the desired resonance [13]. The magneto-electric dipole antennas in [10,11,12,13] achieve wide bandwidth with good gain. However, these structures are complex and multi-layered, which makes it challenging to fabricate and align them precisely. Thus, printed dipole antennas are cost-efficient, compact, and easy to embed in small devices.

The printed dipole antenna results in a narrow bandwidth; however, in [14], a wide bandwidth technique is demonstrated with offset feeding and a tapered dipole structure. The tapered structure on the ground plane and the cross-feed line on the top behave as a balun, improving antenna performance. At mmWave, the angled dipole structure is the most preferred. In [15], the dipole on the ground plane is angled at 45° downwards. A differential slot width extends the dipole. The inverted feed line on the top acts as a balun for the microstrip-to-slot line. In another design [16], the bandwidth of the dipole antenna is improved by adopting the series uniform-spacing and uniform-shape structure. However, the challenge in the MIMO antenna is the surface wave coupling, which can be reduced by defected ground, metamaterial structures [17]; split ring resonators [18]; and parasitic elements [19]. In [20], by optimizing the distance among the MIMO antenna elements, an isolation |S21| of >16 dB was achieved.

The above literature indicates the dearth of printed MIMO dipole antennas. Thus, this article provides a solution with the design of a non-uniformly shaped and non-uniform-spacing dual-element dipole array antenna operating at 28 GHz. The mode excitation of the antenna is studied through characteristic mode theory (CMT). The dual-element dipole array is further expanded to a three-element MIMO antenna to enhance channel capacity and performance. The isolation in the MIMO antenna is achieved through the combination of the proposed metasurface and defected ground structure. The metasurface exhibits a wide stopband at 28 GHz, possessing negative permittivity and zero permeability. The single-element antenna has only the $J_{1}$ mode excited. However, when the antenna is scaled to the three-element MIMO antenna, the structure excites the $J_{1}$, $J_{2}$, and $J_{3}$ modes at 28 GHz. The proposed MIMO antenna achieves a bandwidth of 2.9 GHz with a gain of 6.3 dBi. The isolation is improved from 13 dB to 21 dB due to the metasurface and defected ground structure. Thus, the contribution and novelty of the article are summarized as follows:

(a) Design of a non-uniform-shape and -spacing dual-element dipole array at 28 GHz.
(b) Expansion of the dipole array to a three-element MIMO antenna to enhance channel capacity.
(c) Enhancement of the isolation in a compact MIMO antenna with the design of a metasurface structure.

## 2. Antenna Design

### 2.1. Single-Element Dipole Antenna

The single-element dipole was developed in two stages with the aid of characteristic mode theory (CMT). CMT is a well-known method applied to antenna structures to study the current behavior of the corresponding modes. The required current modes are excited or suppressed through CMT to improve antenna efficiency. Like in the metasurface-based antenna in [21], all the first five modes are significant, in that only the first two modes are degenerative. In contrast, the other modes deteriorate antenna performance. Thus, the antenna structure is modified to suppress the other modes. CMT is also conveniently applied to large structures. In [22], an HF antenna mounting on a large vehicle for bandwidth enhancement is analyzed through CMT by exciting the required modes.

In CMT, the current (J) is induced on the conducting surface (*S*) of the antenna through the incident of tangential electric field $E^{i}$. This induced current J leads to the generation of tangential surface electric field $E^{s}$, where the sum of these tangential fields on the surface is [23,24]

(1) $$E^{s}\left(s\right)+E^{i}\left(s\right)=0\;on\;S$$

By applying the vector function on the surface, the relation of current (J) and induced electric field $E^{s}$ is related to the impedance (Z) as

(2) $$Z\left(J\right)=\left[E^{s}(J)\right]$$

The impedance here is a symmetric operator which has real and imaginary parts:

(3a) $$R=\frac{1}{2}\;\left(Z+Z^{*}\right)$$

(3b) $$X=\frac{1}{2j}\;\left(Z-Z^{*}\right)$$

where, Z=R+jX, with *R* and *X* being the real and imaginary parts of the symmetric operator.

Due to incident $E^{i}$, higher-order surface currents ${(J}_{n})$ with varied magnitudes are inducted at varied frequencies. Current $J_{n}$ with higher significance at the desired frequency radiates the energy from the antenna surface. This is characterized by applying the eigenfunction to Equation (2) [25] as

(4) $$J=\sum_{n=1}^{\infty }\alpha_{n}J_{n}=\sum_{n=1}^{\infty }\frac{\langle J_{n},\;E^{i}\rangle }{1+j\lambda_{n}}J_{n}$$

where *J* is the total current, $\lambda_{n}$ is the eigenvalue, and $\alpha_{n}$ is the weighted coefficient. The parameters of interest in CMT are modal significance (MS) and characteristic angle (CA), which are derived from the eigenvalue ($\lambda_{n}$) as [26]

(5) $${MS}_{n}=\frac{1}{\left|1+j\lambda_{n}\right|}\;$$

(6) $${CA}_{n}=\pi -{tan}^{-1}\left(\lambda_{n}\right)$$

The surface current ($J_{n}$) with zero eigenvalue results in modal significance ${MS}_{n}=1$, indicating that the specific characteristic current mode ${(J}_{n})$ is significant and purely resistive in nature. ${CA}_{n}$ represents the phase difference between $J_{n}$ and its corresponding $E^{s}$. The naturally resonant mode should have a phase close to *π*. For π2<CAn<π orπ<CAn<3π2, the modes ${(J}_{n})$ are inductive or capacitive [27]. In such cases, the antenna is less efficient, as the fed energy dissipates.

**Figure 1:**
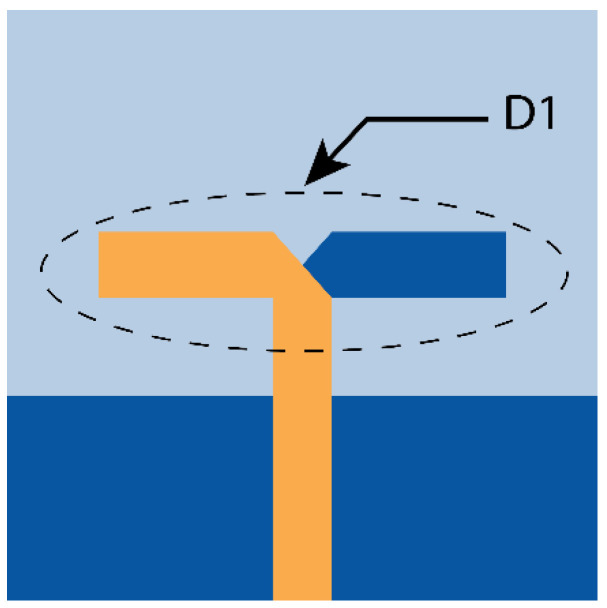
First evolution of dipole antenna with single set of dipole D1.

**Figure 2:**
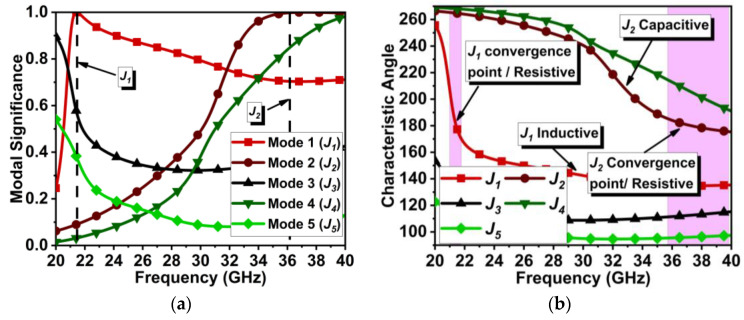
CMT results for antenna design in Figure 1. (**a**) MS*_n_* and (**b**) CA*_n_*.

In our design, the initial antenna has a single set of dipole elements fed by a 50 Ω feed line. One-half element of the dipole is on the top of the Rogers 5880 substrate connected to the feed line, and the other half is at the bottom, connected to the ground plane, similar to [16], unlike non-angular elements. The chosen substrate thickness is 0.254 mm. At first, the single non-angular dipole antenna (D1) ≈λ4 in length is designed and tuned to resonate at 28 GHz (where λ is the wavelength at 28 GHz), as shown in Figure 1. The dipole feed points are trimmed to improve the impedance matching. Applying CMT with five modes to the design in Figure 1 indicates that the Mode 1 $\left(J_{1}\right)$ and Mode 2 $\left(J_{2}\right)$ are significant at 21.5 and 36 GHz. Considering the acceptance threshold of ${MS}_{n}$ > 0.7, both $J_{1}$ and $J_{2}$ have significance over the wide frequency range. However, the intended resonance frequency is 28 GHz, where Mode 1 is just above the threshold, as shown in Figure 2a.

**Figure 3:**
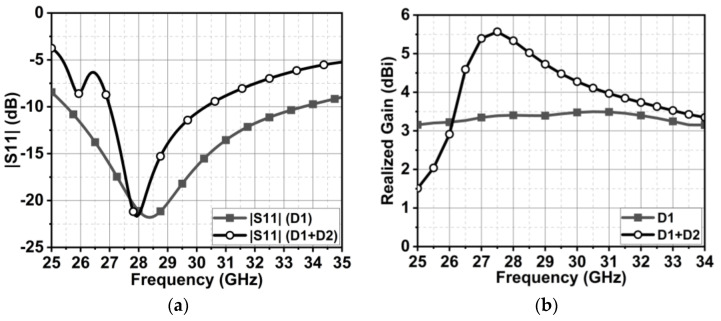
(**a**) is the reflection coefficient curve, and (**b**) is the realized gain comparison of single-element dipole and dual-element dipole antenna (proposed).

The characteristic angle $\left({CA}_{n}\right)$ in Figure 2b indicates that $J_{1}$ has a narrow bandwidth, whereas $J_{2}$ has a wider bandwidth. $J_{1}$ beyond 22 GHz has an increasing inductive effect, which may hinder antenna performance at 28 GHz. With port excitation, the obtained results have a wide bandwidth of 25.5 to 34 GHz, resonating at 28.3 GHz, as displayed in Figure 3a. However, the realized gain of this antenna is low, 3.5 dBi (Figure 3b), and the significance of the $J_{1}$ mode is not appropriate.

**Figure 4:**
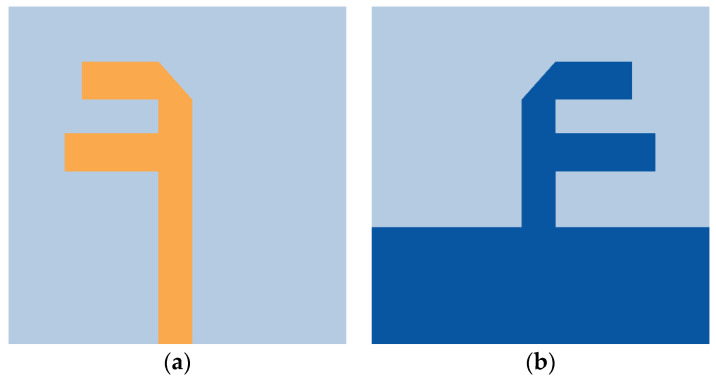
Proposed series-fed, dual-element dipole antenna. (**a**) Top view and (**b**) bottom view.

**Figure 5:**
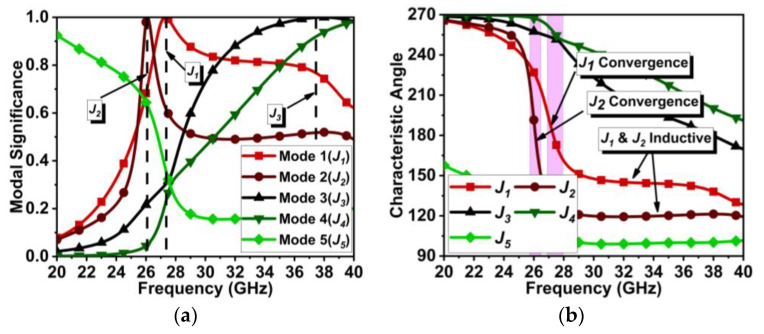
CMT results for antenna design in Figure 4. (**a**) MS*_n_*, and (**b**) CA*_n_*.

**Figure 6:**
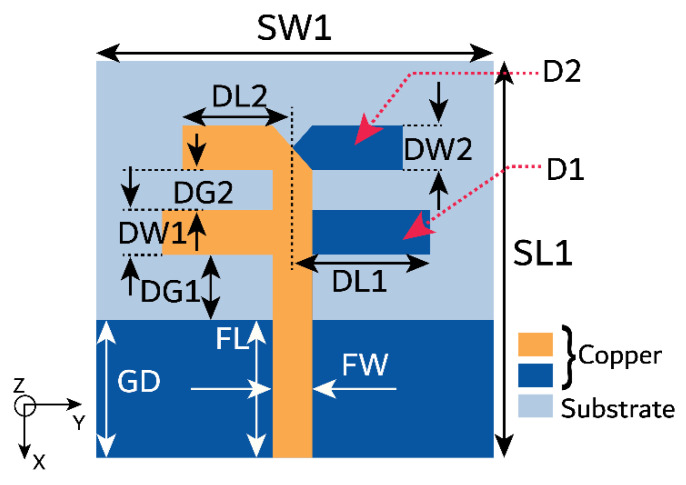
Proposed series-fed, dual-element dipole antenna with dimensions. Dimensions in mm as follows: SW1 = 7, SL1 = 7, FL = 2.5, FW = 0.7, DW1 = 0.8, DW2 = 0.8, DL1 = 2.2, DL2 = 1.75, GD = 2.5, DG1 = 1.2, and DG2 = 0.7.

Further, another dipole antenna (D2) of length λ6 is added, as shown in Figure 4a,b. The two dipole antennas are separated by a distance of approximately λ8. A trimming by an angle of 45° at the feed point of D2 improves the impedance matching at 28 GHz. The addition of D2 to the antenna structure has shifted the characteristic $J_{1}$ mode significance to 27.5 GHz, which is close to the desired frequency. Also, the $J_{2}$ mode is shifted to 26 GHz. $J_{1}$ has wider bandwidth significance, whereas $J_{2}$ has narrow bandwidth significance, as demonstrated in Figure 5a. However, the characteristic angle (${CA}_{n}$) results indicate the extensive phase change over the frequency, leading to a narrow bandwidth in a naturally resonant mode. These modes are inductive for higher frequencies, resulting in lower antenna efficiency. With port excitation, the series-fed, dual-element array dipole antenna has achieved a bandwidth ranging from 27 to 30.4 GHz, as shown in Figure 3a. Though the bandwidth is reduced compared with a single dipole antenna, the realized gain is improved to 5.5 dBi (Figure 3b), and the modes are obtained at the desired frequency. Thus, this also indicates that the beam directivity is increased with a series dual-element dipole array. The proposed dual-element dipole array radiates the energy in the end-fire direction at θ = 90°. Figure 6 shows the proposed antenna with dimensions.

Further, the $J_{2}$ and $J_{3}$ modes are aligned with $J_{1}$, thereby improving antenna performance in the MIMO antenna. Thus, a dual-element dipole array is expanded to a three-element MIMO antenna in the next section.

### 2.2. Three-Element MIMO Antenna

**Figure 7:**
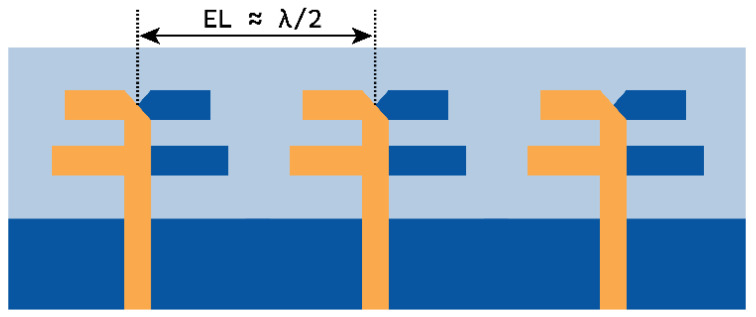
Three-element MIMO antenna with each element antenna composed of a dual-element dipole array.

**Figure 8:**
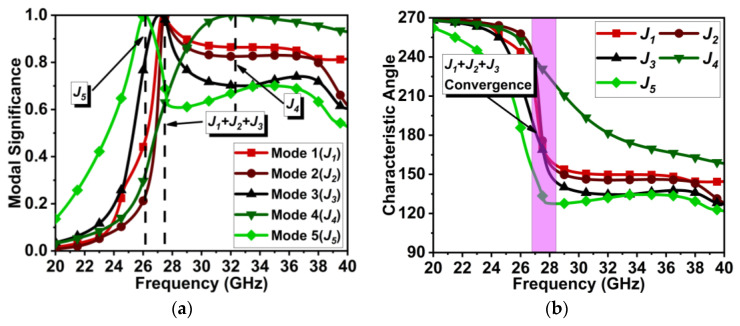
CMT results for MIMO antenna design in Figure 7. (**a**) MS*_n_* and (**b**) CA*_n_*.

The above dual-element dipole array antenna is symmetrically expanded to a three-element MIMO antenna, separated by a distance of approximately λ/2 from the center of the elements, as displayed in Figure 7. The expansion of the dipole array to the MIMO antenna structure has significantly improved the $J_{1}$ to $J_{3}$ modes and the $J_{5}$ mode, as shown in Figure 8a. Here, the $J_{1}$, $J_{2}$, and $J_{3}$ modes almost coincide with each other and are significant at 27.7 GHz. The $J_{5}$ mode is significant at 26.2 GHz. However, the $J_{4}$ mode has significance at 32.1 GHz. Thus, the modes contributing to resonance are $J_{1}+J_{2}+J_{3}$. The characteristic angle in Figure 8b shows the phase convergence of these modes.

**Figure 9:**
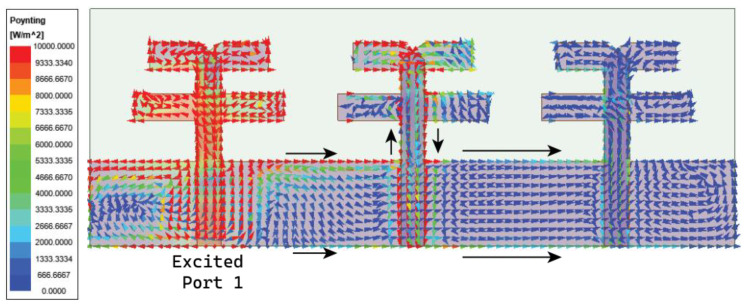
Surface vector current representation in three-element MIMO antenna.

**Figure 10:**
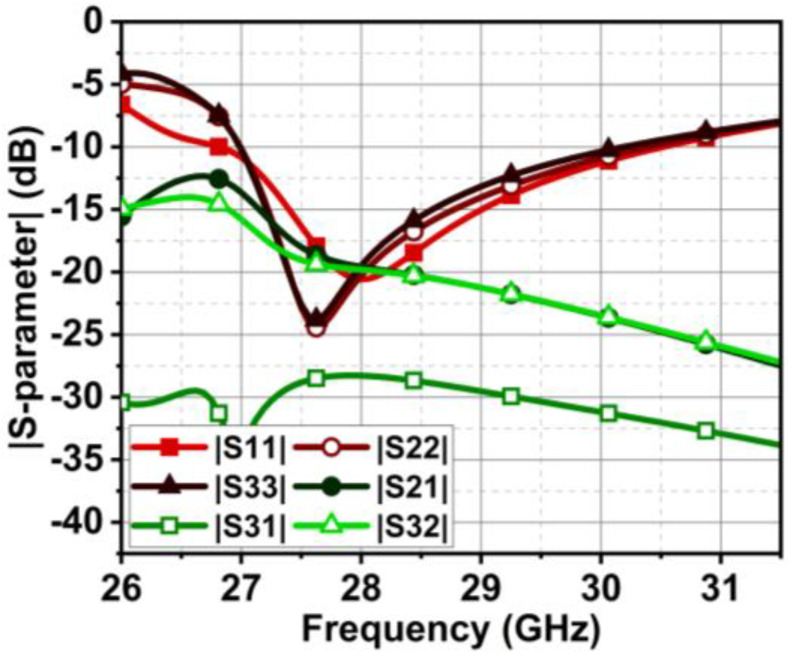
The reflection coefficient |S11| and isolation |S21| curve of the MIMO antenna in Figure 7.

As discussed in the Introduction Section, the major challenge in the MIMO antenna is to suppress the coupling current from adjacent antenna elements. In the case of the angled dipole antenna, the current flows at 45° downwards, causing minimal coupling. However, in our case, the dipole is non-angular, leading to substantial and direct coupling between elements. The coupling in the MIMO antenna is comprehended by observing the Poynting vector current in Figure 9. The black arrows in Figure 9 indicate that the coupling current directly influences the adjacent and subsequent elements. As a result, the isolation |S21| is 13 dB at 27 GHz and gradually increases to 22 dB till 30 GHz, as shown in Figure 10. However, the reflection coefficient is not impeded but may affect overall antenna performance. Thus, a metasurface layer is etched between the elements at top of the substrate to curb the surface wave coupling.

## 3. Metasurface Structure

As observed in the previous section, the coupling current is evident on the adjacent elements. Thus, through the metasurface structure, the coupling effect may be reduced. Consequently, a metamaterial unit cell is designed in this section.

### Unit Cell

**Figure 11:**
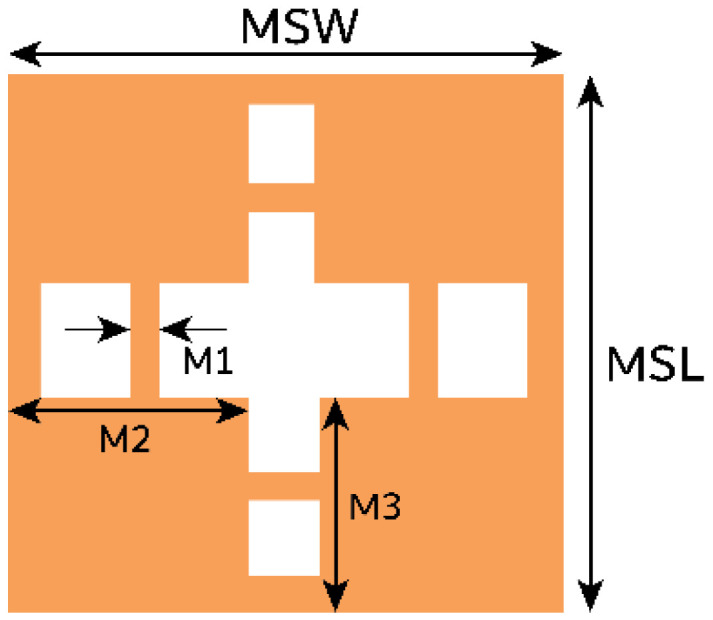
The proposed unit cell has dimensions in mm of MSW = 1, MSL 1, M1 = 0.05, M2 = 0.35, and M3 = 0.4.

**Figure 12:**
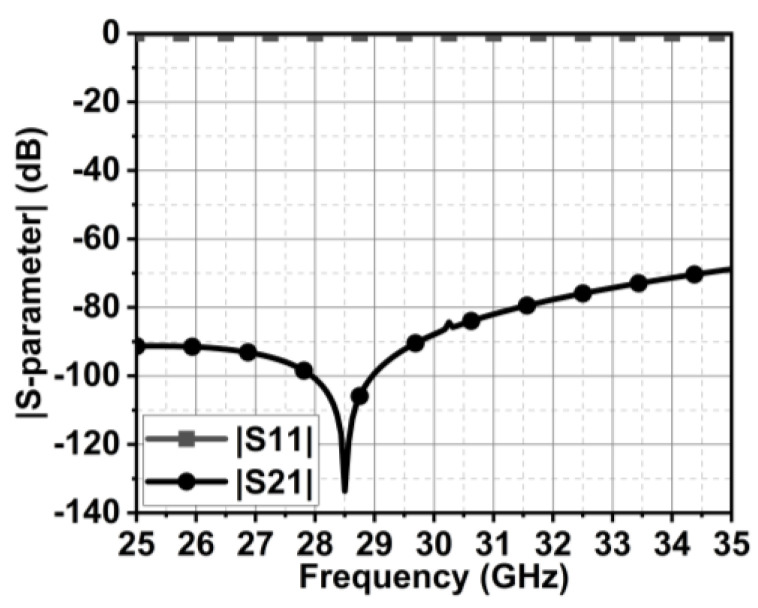
|S-parameter| of proposed unit cell.

The unit cell combines rectangle-shaped structures with a cross-shaped slot in the middle. Further, horizontal and vertical stubs are added to the slot, as shown in Figure 11. The length (MSL) and width (MSW) of the unit are $1\times 1\;{mm}^{2}$. The unit cell exhibits a wide stopband at 28.5 GHz, as illustrated in Figure 12.

The properties of the unit cell, such as its impedance (Z), permittivity ${(\varepsilon }_{eq})$, permeability $\left(\mu_{eq}\right)$, and refractive index (n), are extracted by using Equations (7)–(11) from the |S-parameter| as defined in [28].

(7) $$Z=\pm \sqrt{\frac{{\left(1+S_{11}\right)}^{2}-S_{21}^{2}}{{\left(1-S_{11}\right)}^{2}-S_{21}^{2}}}\;$$

(8) $$e^{jnk_{0}d}=\frac{S_{21}}{1-S_{11}\left(\frac{Z-1}{Z+1}\right)}\;$$

(9) $$n=\frac{1}{k_{0}d}\left[\left\{Im\left[ln\left(e^{jnk_{0}d}\right)\right]+2m\pi \right\}-jRe\left[ln\left(e^{jnk_{0}d}\right)\right]\right]\;$$

where $k_{0}$ and *d* are wavenumbers and the maximum length of the unit cell.

(10) $$\varepsilon_{eq}=\frac{n}{Z}\;$$

(11) $$\mu_{eq}=nZ\;$$

**Figure 13:**
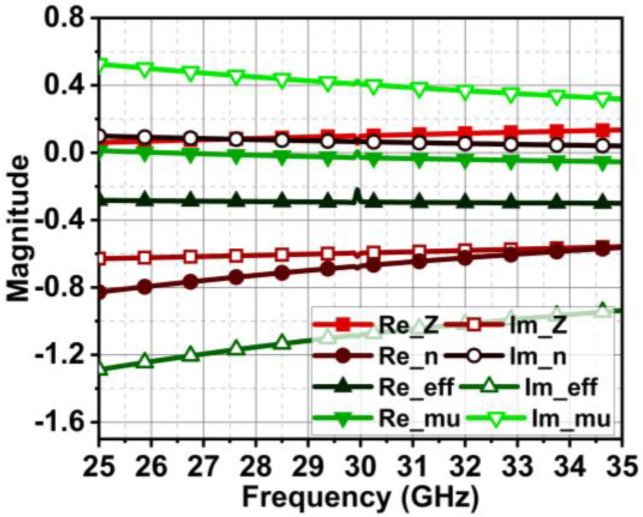
Proposed unit cell properties in terms of its Z, $\varepsilon_{eq}$, $\mu_{eq}$, and n.

**Figure 14:**
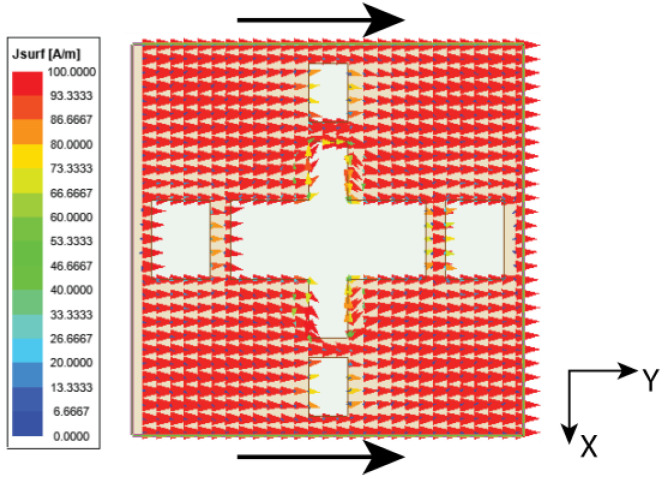
Current flow in metamaterial unit cell due to incident wave.

The proposed unit cell exhibits negative permittivity and zero permeability in the band of interest. Also, it results in a negative refractive index, as displayed in Figure 13. The direction of the current flow in the unit cell due to impinging waves defines its function. Figure 14 illustrates that the current is flowing along the y-axis. For the metasurface to curb the coupling in the MIMO antenna, the coupling current must be redirected upwards or downwards. To achieve this, the unit cell orientation is to be changed by 90°.

## 4. MIMO Antenna with Metasurface

**Figure 15:**
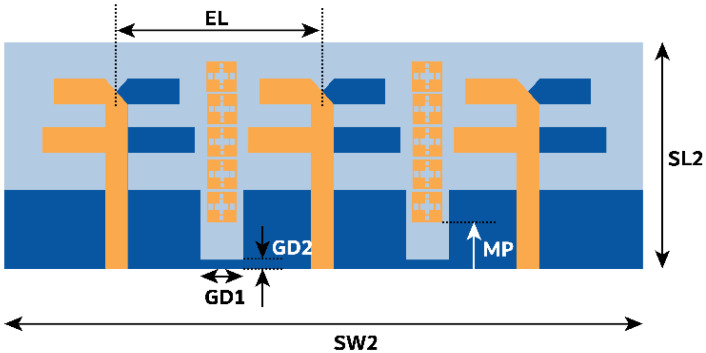
Proposed MIMO antenna with metasurface structure. The dimensions in mm are SL2 = 7, SW2 = 19, EL = 6, GD1 = 1.4, GD2 = 0.25, and MP = 1.3.

**Figure 16:**
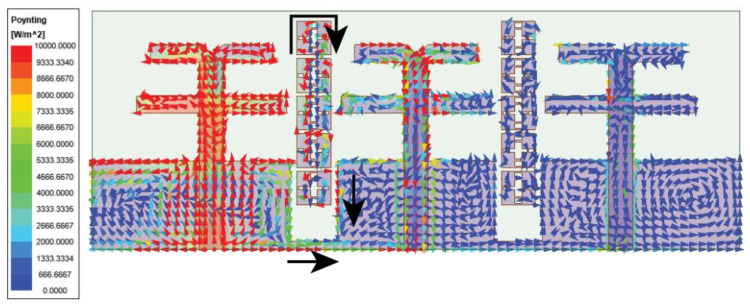
Current vector representation in proposed MIMO antenna with metasurface structure.

**Figure 17:**
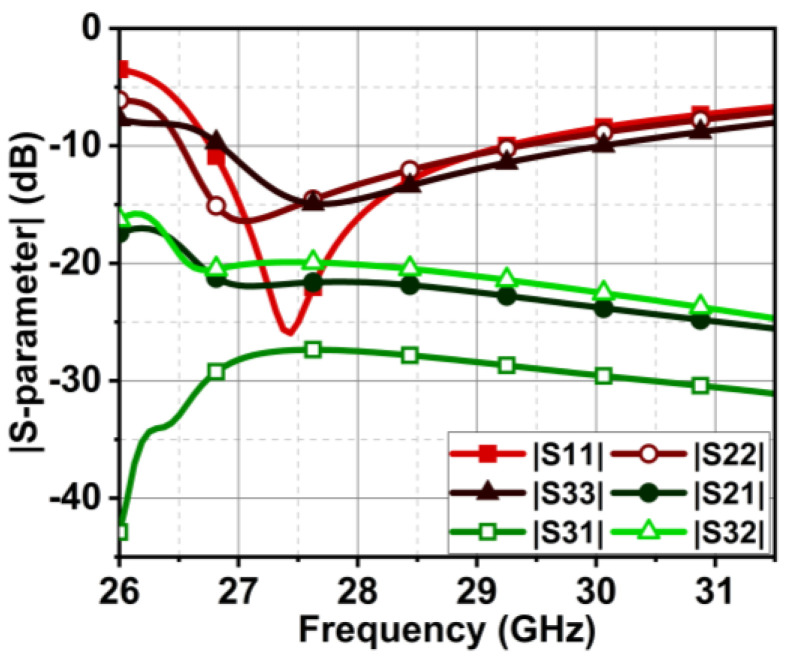
The resulting |S-parameter| of the proposed MIMO antenna with metasurface structure.

**Figure 18:**
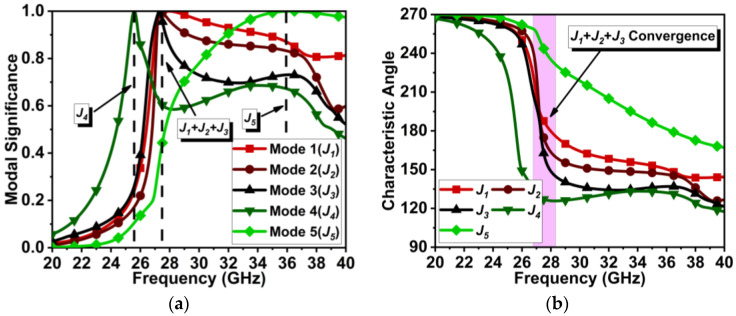
CMT results for MIMO antenna design with metasurface. (**a**) MS*_n_* and (**b**) CA*_n_*.

The above-designed unit cell is orthogonally oriented by 90°, and a series of five cells are arranged between the radiating element at the top of the substrate, as displayed in Figure 15. Further, efficiency in the coupling reduction is achieved by defecting the ground plane. The overall proposed MIMO antenna profile is $0.66\lambda_{0}\times 1.78\lambda_{0}$. The distance between the MIMO antenna elements is λ/2. The decoupling mechanism and functioning of the metasurface can be better understood by considering the Poynting vector in Figure 16. The defected ground path between the MIMO elements causes the forward and reverse current to flow, consequently canceling most of the coupling current. The metasurface also reroutes the coupling current upward (as represented by the black arrow), causing minimal coupling to adjacent elements. Thus, the isolation |S21| is improved to 22 dB, and |S31| reaches 27 dB, almost constant over the bandwidth, as shown in Figure 17. The MIMO antenna results in a bandwidth ranging from 26.75 to 29.7 GHz. CMT investigates the impact of metasurface addition on the excited modes. The modal significance results in Figure 18a specify that the addition of the metasurface has no impact on the three primary modes. This means that the sum of the $J_{1}$, $J_{2}$, and $J_{3}$ modes is still contributing to the resonance. However, the roles of the $J_{4}$ and $J_{5}$ modes are interchanged. The $J_{4}$ mode is now significant at 25.6 GHz, whereas $J_{5}$ mode is significant at 35.7 GHz. $J_{1}$ to $J_{3}$ has zero phase change between $E^{i}$ and $E^{s}$, and as a result, convergence of these at 180° occurs, as shown in Figure 18b.

### 4.1. Impact on Antenna Performance with Change in Number and Location of Metasurface Unit Cells

**Figure 19:**
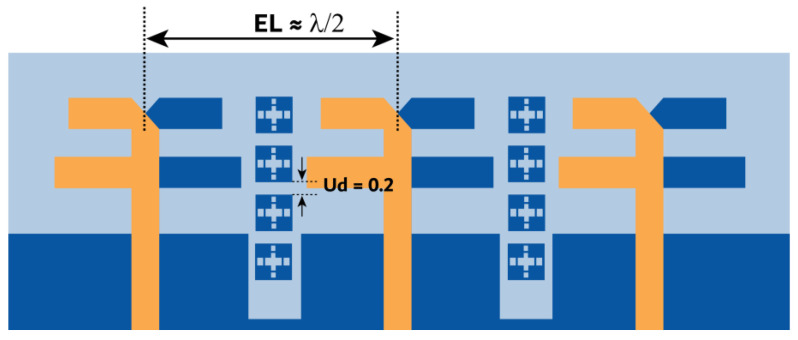
MIMO antenna with an array of four metastructure unit cells at the bottom of the substrate.

**Figure 20:**
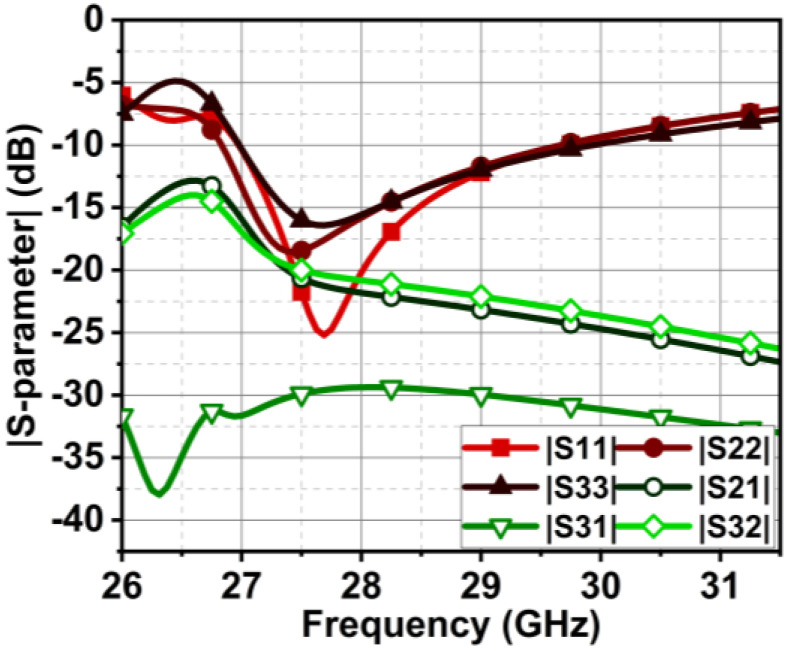
|S-parameter| results of MIMO antenna with four metasurface unit cells at the bottom of the substrate.

The previous section presented the metasurface structure with an array of five unit cells at top of the substrate between the MIMO radiating elements. This arrangement is attained after a rigorous analysis, which is discussed here regarding the number of unit cells and their location and impact on antenna performance. Initially, we begin with the three-unit cells at the bottom of the substrate without changing the ground structure. This arrangement negatively impacts the impedance matching, causing a deviation in the |S-parameter| results. Further, the ground plane is defected, and the number of unit cells is increased to four with a spacing of Ud = 0.2 mm, as shown in Figure 19. The results indicate an improvement in impedance matching; however, isolation |S21| and |S32| at 27 GHz are 15 dB, as displayed in Figure 20.

**Figure 21:**
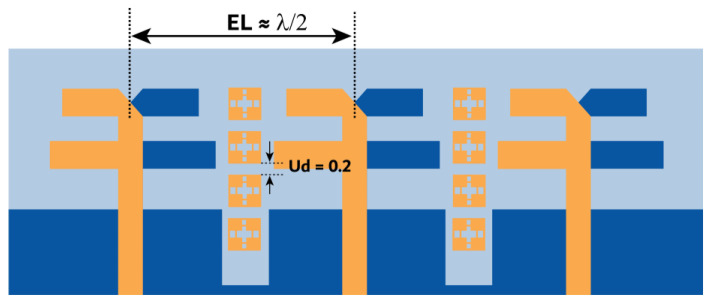
MIMO antenna with an array of four metastructure unit cells at the top of the substrate.

**Figure 22:**
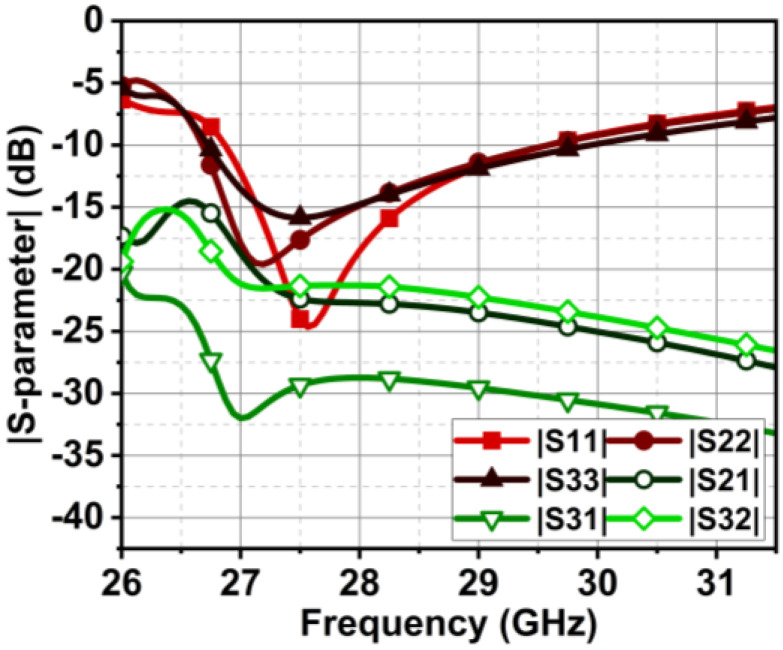
|S-parameter| results of MIMO antenna with four metasurface unit cells at the top of the substrate.

Further, the design is optimized by bringing the metasurface unit cells to the top of the substrate, as shown in Figure 21. In this case, the metasurface unit cells sit in the middle of the MIMO radiating elements, which conceptually makes sense, as it curbs the surface waves between the elements. However, it can be justified with the simulated results. The results in Figure 22 indicate an improvement in isolation at 27 GHz; isolation |S21| and |S32| achieved here are 17.5 and 22 dB. Additionally, from 27.1 GHz onwards, the isolation reaches 22 dB and more. With these satisfactory results, the design is further optimized with five unit cells at the top of the substrate, where the unit cell distance is reduced with Ud = 0.1 mm. The subsequent improvement in the results is shown in the earlier section in Figure 17.

### 4.2. Impact on Antenna Performance with Increase in Inter-element Spacing

In Figure 17, the inter-element spacing is chosen to be very small, that is, EL=λ2=6 mm, whereas the distance from edge to edge is merely 1.6 mm. As a result, the isolation of the proposed antenna is 21 dB at the beginning of the band, which is further improved with an increase in frequency. In this section, the change in MIMO antenna performance is observed by increasing the inter-element distance (EL) to 34λ and 56λ. However, the array of the metasurface is maintained exactly in the middle of the radiating elements, similar to Figure 15.

**Figure 23:**
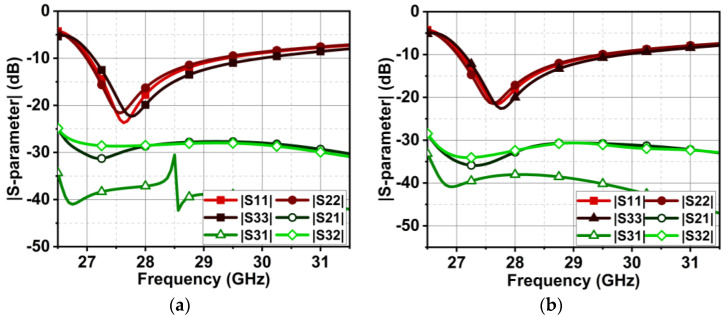
|S-parameter| results of MIMO antenna with five metasurface unit cells at the top of the substrate for an increase in inter-element spacing. (**a**) For EL=34λ and (**b**) for EL=56λ.

For the first case of EL=34λ, the achieved bandwidth is similar to the proposed antenna, that is, from 27 to 29.5 GHz. On the other hand, the isolation |S21| and |S31| are drastically improved to 28 dB, as displayed in Figure 23. The surface wave is a negative function of the exponential function; consequently, the combination of increased distance, metasurface structure, and defected ground have suppressed the surface wave. For EL=56λ, a further suppression of the surface wave is observed, leading to the improved isolation of 31 dB, as shown in Figure 23b.

## 5. Results and Discussion

### 5.1. |S-parameter| and Radiation Pattern

The proposed MIMO antenna comprises three series-fed dual-element array dipoles. The dual-element array improves directivity and the radiation pattern. The proposed MIMO antenna with a metasurface structure can suppress the coupling |S21| to 21 dB at 27 GHz, which improves further with the increase in frequency in the band of interest. On the other hand, the obtained isolation |S31| is > 27 dB, as displayed in Figure 17. However, due to the rerouting of the coupling current by the metasurface structure, a slight discrepancy is observed in the reflection coefficient curves of the MIMO elements. Nonetheless, the bandwidth achieved by all three elements is from 26.75 to 29.7 GHz. The effectiveness of the metasurface structure can be comprehended by observing the |S-parameter| results without the metasurface structure in Figure 10, where the isolation |S21| is lower, 15 dB.

**Figure 24:**
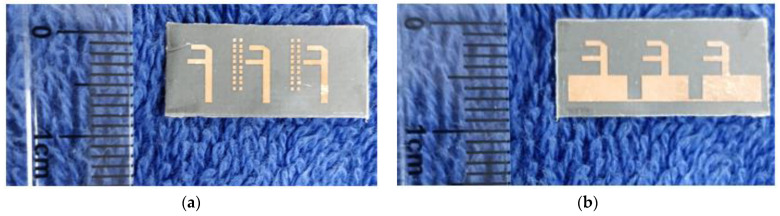
Prototype-fabricated MIMO antenna with metasurface. (**a**) Top view. (**b**) Bottom view.

**Figure 25:**
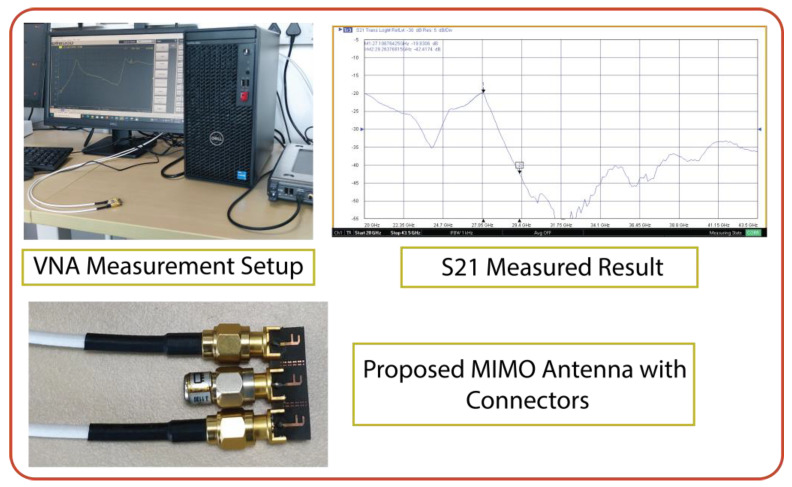
Demonstration of |S-parameter| measurements and setup with VNA for the proposed MIMO antenna interfaced through SMA connectors.

**Figure 26:**
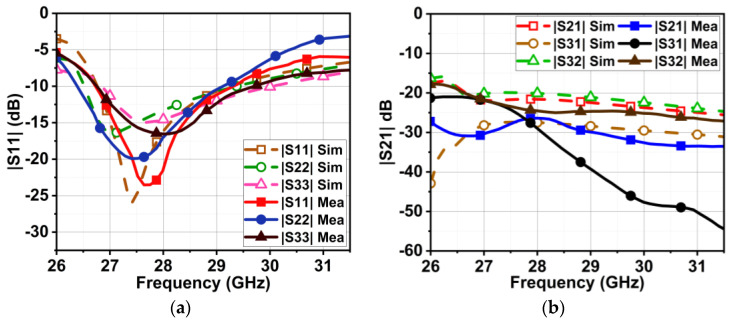
Comparison of simulated and measured |S-parameter| results of proposed MIMO antenna with metasurface. (**a**) Reflection coefficient and (**b**) isolation.

The designed MIMO antenna was prototype-fabricated, as displayed in Figure 24a,b. The connectors used for the measurement were 2.92 mm end launch SMA Johnson connectors, which have an operating range of up to 40 GHz [29]. The designed antenna is highly compact, providing less space for commercial SMA connectors to interface. However, the connectors were interfaced with precision soldering but still provided compact space for VNA cables to connect. Consequently, a slight deviation in results was observed during the measurement. The |S-parameter| measurement setup is demonstrated in Figure 25. The measured bandwidths of |S11|, |S22|, and |S33| were 2.7, 2.6, and 2.9 GHz (26.4–29.1, 26.7–29.3, and 26.7–29.6 GHz, respectively), as displayed in Figure 26a. We measured the following minimum isolation in the bandwidth: |S21|, 26 dB; |S31|, of 32 dB; and |S32|, 21 dB (Figure 26b).

**Figure 27:**
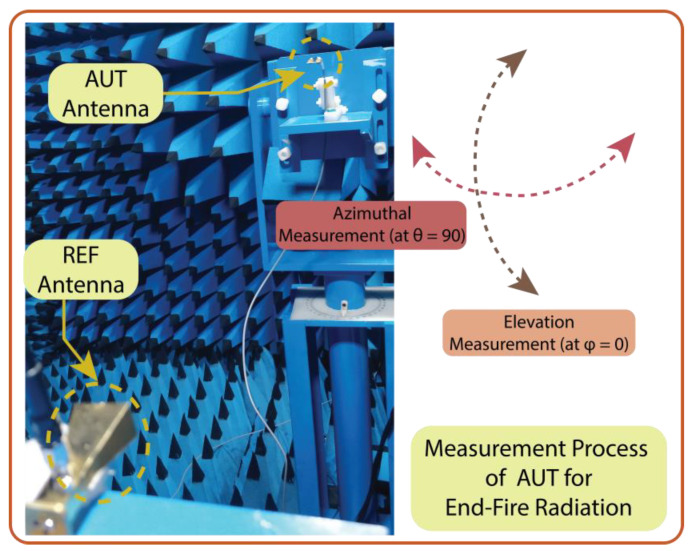
The measurement setup of the radiation pattern in an anechoic chamber.

**Figure 28:**
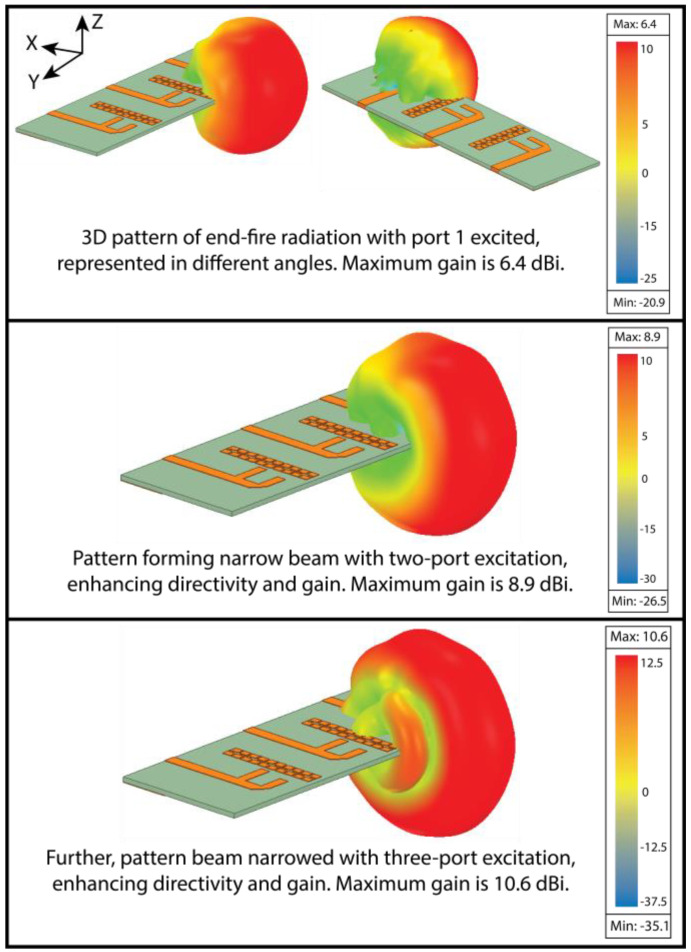
The Three-dimensional plot of the end-fire radiation pattern over the antenna. The figure depicts a pattern for the standalone MIMO antenna and a MIMO antenna with the beam-forming network (when all the elements are fed equal power).

**Figure 29:**
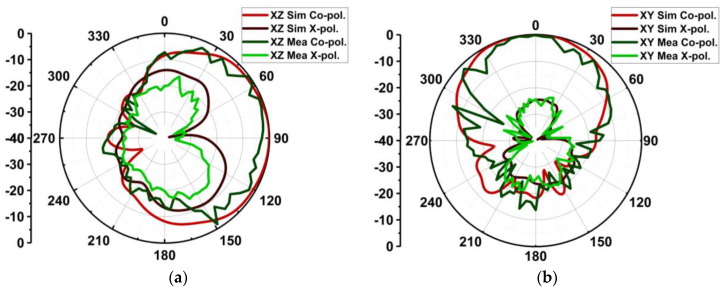
Normalized simulated and measured radiation pattern of proposed MIMO antenna with metasurface on (**a**) XZ plane and (**b**) XY plane.

The measurement setup of radiation in an anechoic chamber is shown in Figure 27. As the proposed structure is a series-fed, dual-element array dipole MIMO antenna, it radiates energy in the end-fire direction. Consequently, the measurement was performed over the XZ plane at ϕ = 0° (elevation plane) and the YZ plane at θ = 90° (azimuthal plane). To better understand the end-fire radiation, the 3D plot over the antenna is presented in Figure 28. In contrast to achieving spatial multiplexing, the gain and beam width of the proposed MIMO antenna were compromised. Consequently, the simulated half-power beamwidths (HPBWs) on the XZ and XY planes were 116° and 82°, respectively, as displayed in Figure 29a. The obtained measured HPBWs were 99° and 49° on the XZ and XY planes, as shown in Figure 29b.

**Figure 30:**
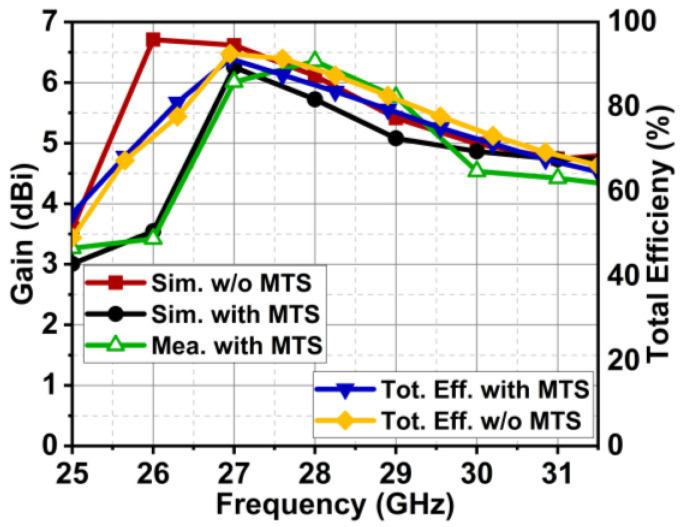
Gain and efficiency plot of MIMO antenna with and without MTS.

The proposed antenna exhibited high cross-polarization of −15 dB on the XZ plane due to the orthogonal E-fields originating from the edges of the feed and dipole. However, lower cross-polarization of −24 dB was achieved on the XY plane. The MIMO antenna without MTS had a maximum gain of 6.7 dBi, whereas a slight reduction of 6.3 dBi was observed with MTS. However, with MTS, the antenna had band-pass characteristics with good gain in the band of interest. On the other hand, the antenna had sharp attenuation, possessing band-stop characteristics outside the region of interest, as shown in Figure 30. The efficiency rates of the MIMO antenna with and without MTS were 91.35% and 92.5%. The MTS surface did not have much of an impact on antenna efficiency.

On the other hand, the proposed MIMO antenna could also achieve a narrow beam pattern with the beam-forming network when all the MIMO elements were fed equal power. As a result, directivity was improved. Consequently, the gain was also improved to 10.6 dBi for the proposed MIMO antenna, as depicted in Figure 28.

### 5.2. Diversity Performance

**Figure 31:**
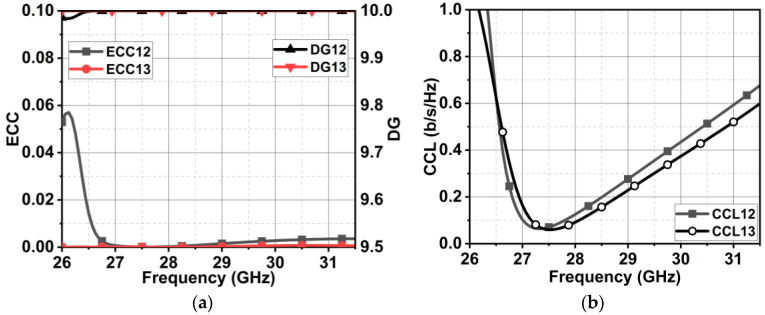
Diversity metrics of proposed MIMO antenna with metasurface. (**a**) ECC and DG and (**b**) CCL.

The proposed MIMO antenna performance is validated with the diversity metrics of envelope correlation coefficient (ECC), diversity gain (DG), and channel capacity loss (CCL). The validation is accomplished by feeding to |S-parameter| the equations as defined in [30]. The ECC resulted in close to zero and DG in approximately 10, as depicted in Figure 31a. The CCL was below 0.4 b/s/Hz, as Figure 31b illustrates. Thus, the proposed MIMO antenna qualifies through diversity metrics.

## 6. Comparative Analysis

The proposed MIMO antenna is compared with the existing planar MIMO antennas in Table 1. The proposed antenna is compact compared with all the other designs in the table. Also, the proposed antenna results in a decent bandwidth compared with [31,32]. The achieved isolation with the aid of MTS is comparable to the other antennas. The proposed antenna has spatial MIMO diversity, which has more advantages than polarized and pattern diversity MIMO antennas. With the beam-forming network, the proposed MIMO antenna can achieve a narrow beam, which the designs in [31,33,34,35] cannot achieve. The proposed MIMO antenna has a good gain of 6.3 dBi compared with [34,36]. The antenna also satisfies the diversity metrics of the MIMO requirement. Also, the antenna has end-fire radiation, which is more suitable for 5G mobile applications. Therefore, the novel contributions of the antenna are compact design, good bandwidth and gain, improved isolation, and the ability to achieve a narrow beam with a beam-forming network. Overall, the proposed MIMO antenna is compact and suitable for future wireless communications.

## 7. Conclusions

This article presented a series-fed, dual-element dipole array, which has end-fire radiation. This dual element enhances the antenna gain from 3 dBi to 5.5 dBi in the end-fire direction. Furthermore, the dual-element dipole array is expanded to a three-element MIMO antenna, where isolation improvement is achieved through the combination of the defected ground plane and metasurface structure. The proposed MIMO antenna results in a bandwidth of 2.9 GHz, resonating at 28 GHz. The structure achieves a minimum isolation of 21 dB and a maximum of 37 dB, with a maximum realized gain of 6.3 dBi. The antenna is also validated through diversity metrics. Thus, the antenna is suitable for future 5G wireless applications.

## Figures and Tables

**Table 1:** Comparison of the proposed MIMO antenna with existing state-of-the-art designs.

Ref.	Dim. in λ	Dim. in mm	No. of Ports	Ant. Type	Res. (GHz)	BW (GHz)	Iso. |S211| in dB	Gain (dBi)	MIMO Div.	MIMO GD	ECC	DG	CCL	Rad.
[31]	2.55λ×5.1λ	20× 40	2-port	Mono. Array	38	37.5–38.5	>40	12	Pol.	NA	$<0.5\times 10^{-4}$	9.9	0.15	BS
[37]	1.4λ×2.43λ	15× 26	3-port	Mono.	28	26.5–34	>25	NA	Sp.	NA	<0.0015	9.9	0.025	BS
[33]	2.81λ×2.81λ	30× 30	4-port	Mono.	28	25–50	>13	NA	Pol.	Dis.	$<2.5\times 10^{-3}$	>9.99	0.21	NA
[32]	3.66λ×2.56λ	43× 30	4-port	Mono. Array	25.5	24.5–26.5	>35	7	Patt.	Con.	<0.0002	>9.99	0.35	BS
[36]	1.11λ×4.41λ	12× 48	4-port	Mono.	27.5/40	24–33/ 48–42	>20	5.7	Patt.	Dis.	<0.00015	>9.99	NA	Omni.
[34]	1.17λ×1.17λ	12.5× 12.5	4-port	Mono.	28	26.5–32	>22	3	Pol.	Con.	<0.27	>9.98	0.25	Omni.
[38]	1.5λ×3.34λ×3.34λ	18× 40× 40	4-port	SRR	25/31	25–26/ 28–33	>30	7.5	3D	Con.	<0.0001	>9.99	<1	EF
[39]	4.32λ×4.32λ	54× 54	8-port	Mono.	24	23.5–27	>25	8.5	Sp. + Pol.	Con.	<0.005	>9.99	NA	BS
[40]	0.91λ×2.53λ	10× 28	2-port	Patch	27	26.5–27/ 39.2–40.5	>31	7	Sp.	Con.	<0.012	>9.99	<0.1	BS
[35]	1.16λ×1.16λ	12.4× 12.4	2-port	Patch	28	26.4–31	>20	6.5	Pol.	Con.	<0.05	>9.95	<0.5	BS
Prop.	0.66λ×1.78λ	7× 19	3-port	DP	28	26.7–29.6	>21	6.3	Sp.	Con.	<0.04	>9.99	<0.3	EF

**Note:** Dim.—dimension; Ant.—antenna; Res.—resonance; BW—bandwidth; Rad.—radiation; Mono.—monopole; SRR—split ring resonator; DP—dipole; Div.—diversity; Pol.—polarization; Sp.—spatial; Patt.—pattern; GD—ground; Dis.—disconnected; Con.—connected; BS—broadside; Omni.—omnidirectional; EF—end fire.

## Data Availability

Data is contained within the article.
